# Stability Profile and Clinical Evaluation of an Innovative Hydrogel Containing Polymeric Micelles as Drug Delivery Systems with Oregano Essential Oil against Fibroepithelial Polyps

**DOI:** 10.3390/ph16070980

**Published:** 2023-07-08

**Authors:** Larisa Bora, Andrada Iftode, Ana Maria Muț, Lavinia Lia Vlaia, Gheorghe-Emilian Olteanu, Delia Muntean, Cristina Adriana Dehelean, Valentina Buda, Georgeta Hermina Coneac, Corina Danciu

**Affiliations:** 1 Department of Pharmacognosy, “Victor Babeș” University of Medicine and Pharmacy, Eftimie Murgu Square, No. 2, 300041 Timisoara, Romania; larisa.bora@umft.ro (L.B.); corina.danciu@umft.ro (C.D.); 2 Research Center for Pharmaco-Toxicological Evaluation, “Victor Babeș” University of Medicine and Pharmacy, Eftimie Murgu Square, No. 2, 300041 Timisoara, Romania; andradaiftode@umft.ro (A.I.); olteanu.gheorghe@umft.ro (G.-E.O.); muntean.delia@umft.ro (D.M.); cadehelean@umft.ro (C.A.D.); buda.valentina@umft.ro (V.B.); 3 Department of Toxicology and Drug Industry, “Victor Babeș” University of Medicine and Pharmacy, Eftimie Murgu Square, No. 2, 300041 Timisoara, Romania; 4 Department II—Pharmaceutical Technology, Formulation and Technology of Drugs Research Center, “Victor Babeș” University of Medicine and Pharmacy, Eftimie Murgu Square, No. 2, 300041 Timisoara, Romania; mut.anamaria@umft.ro (A.M.M.); coneac.georgeta@umft.ro (G.H.C.); 5 Center for Research and Innovation in Personalized Medicine of Respiratory Diseases, “Victor Babeș” University of Medicine and Pharmacy, Eftimie Murgu Square, No. 2, 300041 Timisoara, Romania; 6 Department of Microbiology, “Victor Babeș” University of Medicine and Pharmacy, Eftimie Murgu Square, No. 2, 300041 Timisoara, Romania; 7 Multidisciplinary Research Center on Antimicrobial Resistance, “Victor Babeș” University of Medicine and Pharmacy, Eftimie Murgu Square, No. 2, 300041 Timisoara, Romania; 8 Department of Clinical Pharmacy, Communication in Pharmacy and Pharmaceutical Care, “Victor Babeș” University of Medicine and Pharmacy, Eftimie Murgu Square, No. 2, 300041 Timisoara, Romania

**Keywords:** oregano essential oil, polymeric micelles, hydrogel, fibroepithelial polyps

## Abstract

Skin tags, also known as fibroepithelial polyps (FPs) or acrochordons, are soft, pigmented excrescences, with a prevalence of 50–60% in the population, occurring especially in the fourth decade of life. To date, FPs have been efficiently eliminated using minimum invasive methods such as surgical removal, cauterization, laser irradiation, and cryosurgery. Over-the-counter treatments are also of interest for patients due to their non-invasive character, but their clinical efficiency has not been clearly demonstrated. This study was designed in order to evaluate the efficacy of a modern-pharmaceutical-formulation-type poloxamer-based binary hydrogel, having *Origanum vulgare* L. essential oil (OEO-PbH) as an active ingredient in the management of FPs. The formulation has been shown to possess good qualities in terms of stability and sterility. Non-invasive measurements revealed changes in some physiological skin parameters. An increase in transepidermal water loss (TEWL) and erythema index was noted, while skin surface water content (SWC) decreased during eight weeks of treatment. The macroscopic evaluation revealed that the FPs dried and shrunk after topical treatment with OEO-PbH. Clinically, patients presented a lowering of the number of lesions on the treated area of 20–30% after one month of treatment and around 50% after the second month. Histopathological examination suggests that topical treatment with OEO-PbH may induce histological changes in the epidermis, dermis, and fibrovascular cores of FPs, including a loss of thickness, reduced size and number of blood vessels, and low cellularity. These changes may contribute to the observed reduction in size of FPs after treatment with OEO-PbH.

## 1. Introduction

The skin, also known as the largest organ in the body, is a dynamic and complex anatomic structure. It is divided into three main layers: epidermis, dermis, and subcutaneous tissue [1]. The epidermis consists of epithelial cells, which act as a defense barrier against unwanted agents and are also involved in the epithelialization process [2]. In addition, the stratum corneum is the outermost layer of the epidermis, a brick-and-mortar system composed of mature keratinocytes which act as a permeability barrier, preventing the excessive loss of transepidermal water and entering of exogenous agents [3]. Furthermore, the dermis is the underlying layer of the skin, connected with the epidermis through the dermal–epidermal junction. The dermis is formed mainly of collagen and elastin fibers, which support the epidermal layer of the skin, and fibroblasts [4].

While the skin is permanently in direct contact with exogenous factors which can imbalance its function, such as pollutants, irritants, allergens, and infectious agents, it is mandatory to maintain a solid skin barrier function for healthy functioning of the skin [5]. In addition, endogenous factors (aging, genetic predisposition, sebum and sweat secretion, and skin hydration) can also have an important impact on the pathogenesis of cutaneous disorders. Fluctuations and changes in the skin pH, which is normally slightly acidic (4.7–5.7), are often connected with the occurrence of inflammatory (atopic dermatitis, contact dermatitis) and infectious diseases (bacterial or fungal skin infections), mainly due to skin barrier dysfunction [6,7].

FPs, also known as acrochordons or commonly known as skin tags, have a high prevalence rate (approximatively 50–60%) within the adult population. These soft and pigmented excrescences tend to occur the most frequently during the fourth decade of life [8]. Microscopically, FPs are characterized by a central core of loose fibrocollagenous tissue, which is surrounded by a thin layer of epidermis. The fibrous core is composed of numerous thin, elongated collagen fibers, interspersed with small blood vessels and sparse adipose tissue. The overlying epidermis may be hyperplastic, and may also contain keratin-filled cysts and occasional pigment in basal epidermal keratinocytes. FPs are attached to the skin via a pedunculated thin stalk. They develop markedly in naturally folded areas of the skin, such as neck, axillary, inguinal, breast, and eyelid surfaces [9,10]. Although FPs are harmless, they can be really disturbing for the patient due to inaesthetic appearance and constant friction, which cause irritation and itching symptoms and even bleeding. They appear in both women and men, and increase in number and vary in size with age [8]. Moreover, the appearance of acrochordons is significantly related to obesity, diabetes, metabolic syndrome, hormonal imbalance, and genetic predisposition [11]. 

One of the possible mechanisms involved in the occurrence of FPs entails a hormonal disequilibrium, e.g., increased levels of estrogens and androgens, especially in overweight people. This imbalance can lead to a hyperproliferation of keratinocytes and fibroblasts due to the high affinity of α- and β-estradiol for these cells [12]. Furthermore, insulin-like growth factor-1 (IGF-1), stimulated by hyperinsulinemia, can also be involved in the etiology of FPs due to a local upregulation of this gene and increased production of the IGF-1 protein. IGF-1 stimulates fibroblast proliferation, which further cascades a hyperplasia of keratinocytes with the occurrence of hyperkeratosis and papillomatosis [13,14]. FPs are also strongly associated with a certain risk of development of metabolic syndrome characterized by the co-occurrence of dyslipidemia, hypertension, obesity, and insulin resistance, specifically in the case of patients with low HDL and high waist circumference [15,16].

Currently, the effective removal of FPs involves minimally invasive techniques such as cryosurgery, cauterization, surgical excision, and laser therapy. While non-invasive treatments available over the counter have piqued the interest of patients, their clinical efficacy has not been plainly established yet [17]. 

Nowadays, there is a growing demand for botanical and natural dermato-cosmetic products due to the current “back to nature” trend. Therefore, natural origin compounds are more likely to be used in cosmetics, cosmeceuticals, or pharmaceuticals to the detriment of synthetic substances [18,19]. Whilst the terms “cosmetics” and “pharmaceuticals” are well defined, the term “cosmeceuticals” may be a little confusing for the large population. Cosmeceutical products possess the ability to produce significant changes at the skin level, by resembling dermatological products. On the other hand, cosmeceuticals are not subject to the same regulatory, safety and efficacy studies as dermatological products, also known as pharmaceuticals, and can be purchased without a medical prescription [20,21]. Since consumers are constantly searching for diverse, novel and improved skin care products, the cosmeceutical marketplace can offer high-quality products delivering various skin benefits such as antioxidant, anti-inflammatory, lightening, anti-aging, moisturizing, and exfoliating effects [22].

Plant-derived active phytochemicals have sparked curiosity ever since ancient times and are at present investigated in depth due to their various applications in medicine, veterinary medicine, the food industry, cosmetics, and natural dye production [23,24]. In this light, aromatic plants are considered to be a versatile class of plants that are primarily used for their fragrance and taste as well as for their beneficial effects in a wide range of pathologies due to their complex phytochemical composition [25]. Essential oils (EOs) are variable mixtures of secondary metabolites that include phenylpropenes and terpenes, responsible for their intense fragrance. Monoterpenes and sesquiterpenes, the main phytochemicals of volatile oils, are produced in various plant organs, including bulbs, flowers, seeds, leaves, twigs, bark, wood, fruits, and roots [26]. Consequently, it is well known that the strong odor of aromatic plants is associated with the high content of EOs [27]. Thus, EOs have gained popularity in the dermato-cosmetic and perfumery industries due to their strong fragrant capacity, complex phytochemical profile, and the natural marketing trend [18]. 

The actual tendency of researchers is to develop novel and modern plant-based cosmeceutical formulations by means of micro- and nanotechnology to overcome some physio-chemical limitations of natural compounds. EOs are characterized by an irritant and instable profile, hence the need for delivering efficient drug delivery systems. Both micro- and nanoencapsulation methods offer controlled release of active components, enhance the biological activity, protect the volatile compounds from degradation and evaporation, and improve the overall appearance of the finite product [28,29]. Furthermore, a wide palette of EOs incorporated into various dermato-cosmetic formulations are currently being studied in order to assess their pharmacological potential [19]. In vitro assays have demonstrated the anti-bacterial and anti-fungal activities of a multitude of formulations containing EOs, namely *Lamiaceae* species (*Lavandula sp., Thymus sp., Origanum sp., Rosmarinus sp.*), *Melaleuca alternifolia* (Maiden and Betche) Cheel, and *Cinnamomum zeylanicum* Blume, thus being choices for anti-microbial agents and preservatives [30,31,32,33,34]. Focusing on in vivo assessments of animal and human subjects, formulations with EOs have exhibited impressive bioactivities such as anti-Malassezia effect (*Citrus aurantium* L., *Lavandula officinalis * Mill., *Origanum sp., Mentha x piperita* L., *Helichrysum italicum* (Roth) G. Don) [35], mosquito repellent effect (*Cymbopogon winterianus* Jowitt, *Piper aduncum* L.) [36,37], UV-B protection (*Calendula officinalis* L.) [38], anti-psoriatic activity (*Kunzea* oil) [39], lightening effect and hair growth inhibition (*Curcuma aeruginosa* L.) [40], anti-acne potential (*Myrtus communis* L., *Origanum vulgare* L.) [41], and wound healing capacity (*Origanum* sp., *Salvia triloba* L.) [42].

Oregano (*Origanum vulgare* L.) is a versatile member of the *Lamiaceae* family owing to both its culinary and medicinal attributes. It is located and cultivated mostly in the Mediterranean region, but also in several areas of Europe, Asia, North Africa, and America with mild, temperate climates [43]. In terms of chemical composition, thymol and carvacrol are the most abundant phenolic monoterpenes in *Origanum vulgare* L. essential oil (OEO), especially in the Greek one, accompanied by their precursors (*p*-cymene and *γ*-terpinene) [44]. *O. vulgare* L. has been added to Europe’s List of Priority Species due to its unique biological characteristics and economic significance [45]. The essential oil extracted from *O. vulgare* L. has been intensively studied for novel therapeutic applications in the dermato-cosmetic field [46]. In this light, effects such as anti-acne, anti-aging, and wound healing have been strongly sustained by in vitro and in vivo studies reviewed in one of our previous articles [47]. In addition, some attempts have been made recently to use OEO as an active ingredient in various colloidal drug carriers. The results presented in the studies are encouraging, suggesting that these nanocarriers enhance the bioavailability and thus the bioactivity of oregano essential oil [48]. 

To improve the solubility and the bioactivity of OEO, we previously developed a modern pharmaceutical formulation, namely a poloxamer-based binary hydrogel (OEO-PbH) containing polymeric micelles as carriers for OEO. In comparison with other conventional semisolid topical vehicles, this polymeric hydrogel is superior due to its main components, namely the two poloxamers, which present the following advantageous characteristics: (i) they are biocompatible, non-toxic, and non-irritant, and therefore ranked as GRAS (generally recognized as safe) by the FDA and included with individual monographs in the European and US Pharmacopoeia; (ii) in aqueous medium, at room temperature, they form gels with longer residence time on the skin, resulting in higher OEO local concentrations available for therapeutic effect; (iii) they are considered to be efficient penetration enhancers; (iv) they act as solubilizers for poorly hydrosoluble active ingredients (including OEO), encapsulating them in polymeric micelles, thus achieving their better safety profile when topically applied [49,50]. Also, this polymeric hydrogel could be considered as an easily accessible and user-friendly over-the-counter preparation, as a non-invasive treatment approach, in comparison with the abovementioned minimum invasive methods currently used to eliminate skin tags. This formulation was physicochemically characterized just after preparation, in terms of macroscopic appearance, pH, particle size, and consistency (indicated as penetration degree and spreadability as consistency-related parameters) [51]. In vitro and in ovo studies were also conducted to evaluate the biological activities of OEO-PbH on the human keratinocyte (HaCaT) cell line [51] and on chick chorioallantoic membrane (CAM) [50], respectively. Because of the fact that OEO-PbH formulation contains an active ingredient such as essential oil and its vehicle is a polymeric-micelle-based hydrogel, which is a supramolecular self-assembly, more complex than a single-phase gel, the physicochemical characterization of this formulation is usually complemented with in vitro data referring to its stability over time, under storage conditions [52,53].

Therefore, in this study, several attributes of OEO-PbH, namely its susceptibility to microbial contamination and stability over time, were investigated to complete the quality control tests previously performed and reported. Moreover, a clinical study of this innovative hydrogel was carried out involving both non-invasive methods as well as histological evaluation.

## 2. Results

### 2.1. Long-Term Stability Evaluation of the Experimental OEO-PbH Formulation

Considering the recommendations of the regulatory authorities presented in pharmacopoeias and national formularies for extemporaneous compounding formulations [54], the long-term stability of experimental OEO-PbH was studied for 6 months under appropriate storage conditions (room temperature and amber tightly closed container). At pre-established times, we tested several quantifiable properties that could change during the storage period, namely organoleptic properties, pH, OEO content, and consistency. The experimental OEO-PbH formulation was considered to be stable if its physical properties did not change significantly and contained more than 90% OEO. 

**Figure 1:**
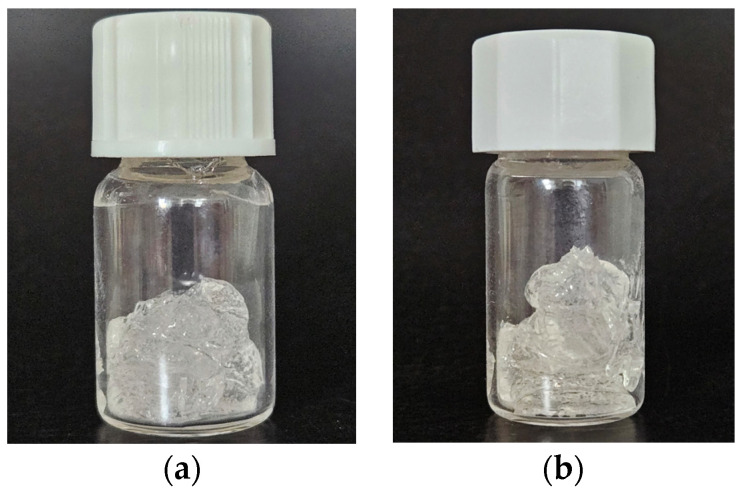
Macroscopic appearance of the experimental OEO-loaded poloxamer binary hydrogel: (**a**) 24 h after preparation and (**b**) 180 days after preparation.

As was previously reported [51], at 24 h after preparation, the OEO-PbH formulation was homogenous and presented a clear, transparent, yellowish appearance, with a specific odor of oregano essential oil. When subjected to visual examination at different times over a 6 month period (days 7, 14, 28, 60, 90, and 180), the OEO-PbH formulation stored at 25 °C did not change in physical appearance, keeping its stable gel structure and the homogenous, clear, transparent, and yellowish appearance, without any change in its specific odor, and without visible phase separation (Figure 1).

Along with the evaluation of organoleptic properties, the study of pH profile of a formulation over time provides information about the chemical stability of the preparation, with it being possible that the variations in this parameter correlate with decomposition processes of the vehicle components and/or the active ingredient.

**Figure 2:**
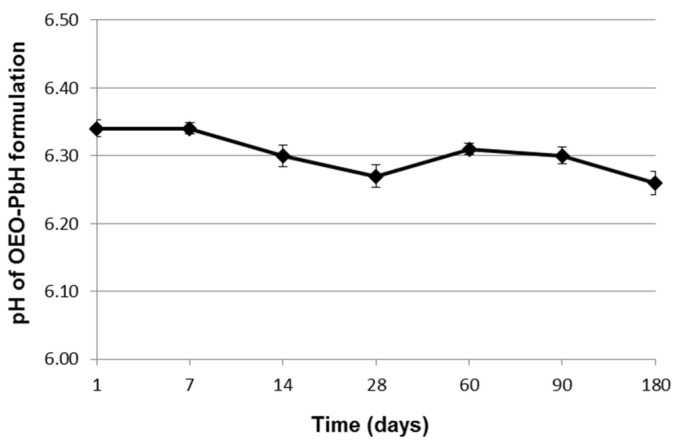
pH of the experimental OEO-loaded poloxamer binary hydrogel during the stability testing period (day 1 to day 180). Data are presented as mean ± standard deviation (SD).

After 24 h, OEO-PbH showed a very slightly acidic pH (6.34 ± 0.05), attributed to the two poloxamers used as gelling agents [51]. Figure 2 presents the pH profile of the OEO-PbH formulation over 6 months of storage at room temperature, indicating no significant differences between the pH values measured at different times. 

**Figure 3:**
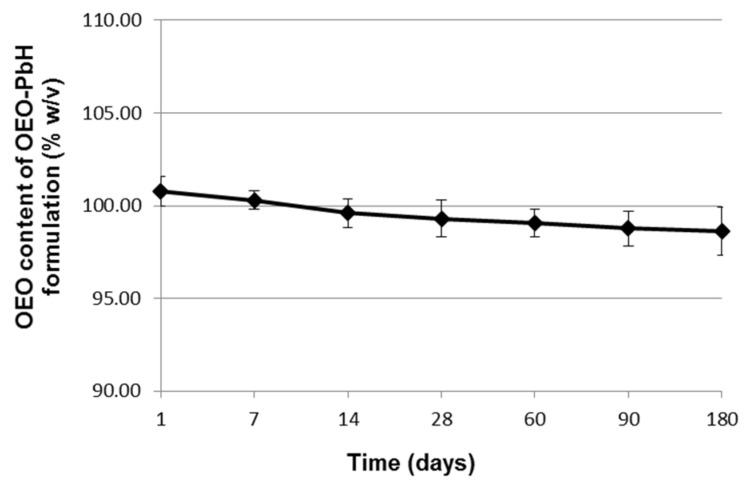
OEO content of the experimental OEO-loaded poloxamer binary hydrogel during the stability testing period (day 1 to day 180). Data are presented as mean ± standard deviation (SD).

The results of OEO content analysis showed that the OEO concentration in the studied poloxamer binary hydrogel remained above 98% (*w*/*v*) throughout the 6 months of stability testing at room temperature and varied within a narrow range (from 98.60 ± 1.29% to 100.80 ± 0.80%) (Figure 3). 

**Figure 4:**
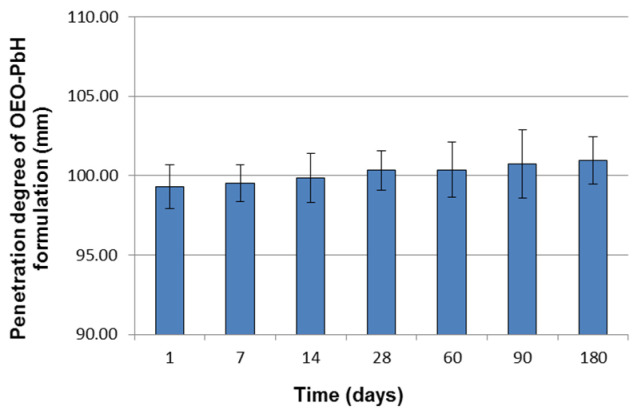
Penetration degree (in mm) of OEO-PbH formulation as a function of storage time (days). Data are presented as mean ± standard deviation (SD).

The penetration degree values of OEO-PbH that resulted from the penetrometry measurements during the long-term stability study are presented in Figure 4.

It can be observed that these values increased insignificantly over time, ranging between 99.30 ± 1.36 mm at 24 h after preparation and 100.97 ± 1.48 mm after 6 months (Figure 4), thus indicating no considerable changes in the hydrogel hardness.

**Figure 5:**
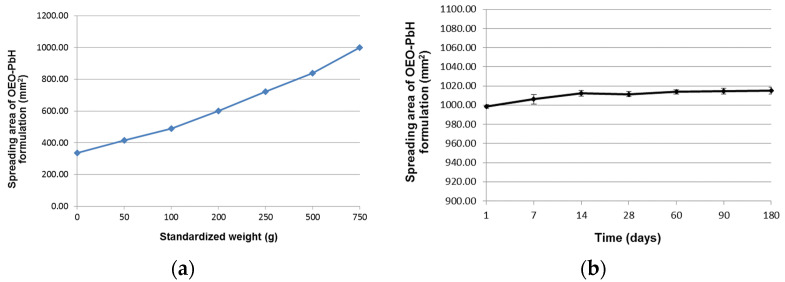
Spreadability profile of experimental OEO-PbH formulation: (**a**) as a function of applied weight (g) at room temperature on day 1 after preparation and (**b**) as a function of storage time (days) after applying the highest standardized weight.

The results of the spreadability test performed at room temperature 24 h after the preparation of the experimental OEO containing hydrogel were presented in our previously published study [51]. It was observed that the spreadability of the formulation increased progressively with the added standardized weights (Figure 5a), revealing its relative ease of being spread on the skin. 

When the spreadability test was repeated for the remaining time points (days 7, 14, 28, 60, 90, and 180), similar and almost overlapping extensiometric profiles were obtained with the one recorded on day 1 after preparation. For the clarity of the graphic representation, in Figure 5b, we only show the values of the spreading areas calculated after adding the highest standardized weight at the respective remaining time points.

### 2.2. Microbiological Assessment of OEO-PbH

The total count of colony-forming units (CFUs) was determined by counting the colonies that developed on agar plates, with values below 300 colonies/plate for bacteria and below 100 colonies/plate for fungi being considered to be microbiologically satisfactory.

To detect bacterial growth and establish the total count of CFU/g sample, the change in color of the medium was followed, depending on the number of test tubes and the dilutions. All of the tested samples were negative. As described in the methodology, Salmonella spp., Escherichia coli, Pseudomonas aeruginosa, and Staphylococcus aureus were absent. The preservative efficacy test showed that none of the microbial strains were isolated from the 48 h samples, with the inoculated culture media remaining sterile.

### 2.3. Erythema Index, TEWL, and -SWC Assessment

**Figure 6:**
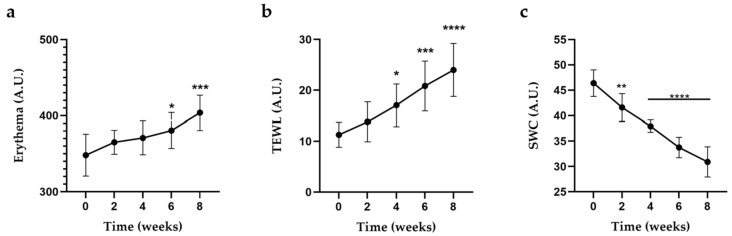
(**a**) Evolution of erythema index, (**b**) TEWL, and (**c**) SWC throughout the 8 weeks of topical application of OEO-PbH. Data are presented as mean ± standard deviation (SD). Measurement analysis was performed using one-way ANOVA and Dunnett’s multiple comparison test (* *p* < 0.05; ** *p* < 0.01; *** *p* < 0.001; **** *p* < 0.0001).

The effect of OEO-PbH on FPs was evaluated along the eight weeks of topical application by means of mexametric, corneometric, and tewametric measurements, expressed in arbitrary units (A.Us.). Compared to the normal values registered at time 0, the erythema index showed a slow growing trend (Figure 6a). It can be observed that the FP irritation level increased exponentially in a time-dependent manner, with statistical significance at weeks six and eight of treatment. The same trend can also be observed in Figure 6b regarding the TEWL values. There was a significant increase in the TEWL at the level of FP during the treatment with OEO-PbH. The results of corneometric measurements are shown in Figure 6c. After treatment with OEO-PbH, a significant decrease in SWC was noted, thus showing the skin hydration of papillomas even from the first few weeks of application.

### 2.4. Histopathological Examination

The FPs that were excised at the initial dermatology consultation without any prior treatment were processed for use as a benchmark for histological changes induced via the application of the topical OEO-PbH. A microscopic examination of these FPs revealed a typical histological composition with a central core of loose fibrocollagenous tissue surrounded by a thin layer of epidermis.

After one month of topical treatment with OEO-PbH, the microscopic examination of the FPs revealed a thinner epidermis in most cases, as well as a more edematous dermis and a marked increase in inflammatory cells at the same level. These histological changes suggest that OEO-PbH may have anti-inflammatory effects and may affect the thickness of the epidermis.

After two months of topical treatment with OEO-PbH, the microscopic examination of the FPs revealed that the fibrovascular cores presented the most obvious change with a loss of thickness, reduced size and number of blood vessels, and low cellularity. These changes suggest that OEO-PbH may influence the fibrous core of FPs, leading to a reduction in size and changes in the blood supply to the FPs.

**Figure 7:**
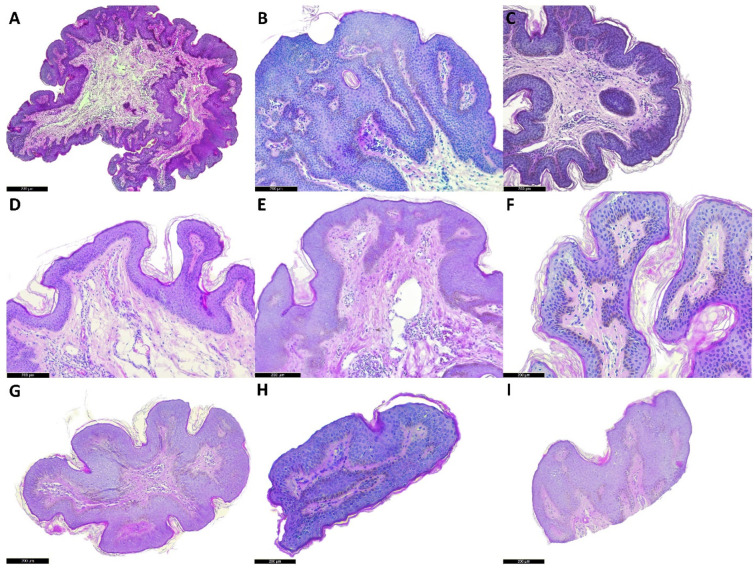
Representative cases from the study cohort. (**A**–**C**) are microscopic examples (hematoxylin and eosin (H&E) stain) of FPs excised without prior treatment. These were processed for use as a benchmark for histological changes induced via the application of the topical OEO-PbH. (**D**–**F**) are microscopic examples (H&E stain) of FPs that were excised after 1 month of topical treatment with OEO-PbH. After 1 month of treatment, a thinner epidermis could be observed in all 3 example cases, as well as a more edematous dermis and a marked increase in inflammatory cells at the same level. (**G**–**I**) are microscopic examples (H&E stain) of FPs that were excised after 2 months of topical treatment with OEO-PbH. After 2 months of treatment with OEO-PbH, FPs that were grossly processed were reduced in size and, as seen microscopically, the fibrovascular cores presented the most obvious change with loss of thickness, reduced size and number of blood vessels, and low cellularity.

Overall, the microscopic examination of FPs at different time points suggests that topical treatment with OEO-PbH may induce histological changes in FPs, including changes in the epidermis, dermis, and fibrovascular cores (Figure 7). These changes may contribute to the observed reduction in size of FPs after treatment with OEO-PbH.

## 3. Discussion

The present study expands on the prior investigations conducted by our research group. Using gas chromatography–mass spectrometry, we previously characterized the phytochemistry of OEO extracted via hydrodistillation from the aerial parts of Romanian *O. vulgare* var. *vulgare*. Forty-three volatile compounds were identified and quantified, with carvacrol and thymol as the main components. Furthermore, OEO-loaded poloxamer binary hydrogel was prepared and evaluated in terms of physicochemical properties (pH, spreadability, consistency, polydispersity index, particle size, and zeta potential). The formulation exhibited good skin compatibility in terms of consistency and spreadability. A biological evaluation of OEO-PbH was also conducted. Both OEO and OEO-PbH presented significant anti-microbial activity. Furthermore, an in vitro assessment of HaCaT human keratinocytes on human dendritic cells was conducted. OEO-PbH demonstrated anti-proliferative, anti-migratory, and pro-apoptotic effects on HaCaT cells, while no injurious effect on dendritic cells was detected. In addition, the formulation determined a decrease in inflammatory cytokines (IL-6, IL-23, and TNF-α) without affecting IL-10 [51]. 

The next step in our comprehensive investigation involved a morphological characterization of OEO-PbH by means of scanning electron microscopy analysis. A regular aspect was observed after OEO encapsulation. In vitro release and skin permeation studies showed a sustained release of OEO through synthetic and pig ear membranes. Moreover, we conducted an extensive in ovo assessment on CAM in order to establish the potential effects of OEO-PbH on angiogenesis and the biocompatibility of the tested formulation. OEO-PbH was well tolerated and did not present an irritative effect on CAM. Additionally, it showed an anti-angiogenic effect on CAM, especially on the blood vessels [50]. 

Stability testing is an important assay in the quality control of a pharmaceutical product, since it produces evidence on how various environmental factors (e.g., temperature, humidity, and light) can influence and, consequently, determine variations in the quality of a pharmaceutical preparation over time [55]. In the case of complex pharmaceutical semisolids containing multiple phases, such as polymeric-micelle-based hydrogels, increased temperature can modify their phase transitions (gelling behavior), micelle formation process, and phase distribution [56]. Furthermore, the long-term stability study of topical products based on nanocarriers, including polymeric micelles, loaded with essential oils provides evidence on carrier effectiveness to protect the volatile compounds from degradation and evaporation under the influence of temperature, light, and/or air.

The findings of pH determination, correlated with those of visual examination, suggest three beneficial attributes of the studied experimental formulation: (1) chemical stability, as there was no indication that the degradation of poloxamers and essential oil components occurred over the 6 month period; (2) thermodynamic stability of polymeric micelles and consequently the maintenance of their ability to solubilize the OEO, as there was no significant change in the formulation pH that could have destabilized the micelles [57]; and (3) no irritability and tolerance problems when applied to the skin, since the pH values remained near the range of normal skin pH [58].

The results of the OEO content assay showed that the OEO concentration in the formulation was kept above 90% at 25 °C for 6 months, complying with the specification of current pharmacopoeias and revealing the uniformity of active ingredient content and its chemical stability under the storage conditions, with this last-mentioned attribute also being confirmed by the pH study. Consequently, one can consider that no loss in potency of OEO occurred at the end of the stability test [59].

For pharmaceutical semisolid preparations, consistency, or structural strength, assessed through its two components, hardness and spreadability, is an important rheological feature which can significantly affect several characteristics, including stability, homogeneity of incorporated active ingredient, bioadhesivity, and finally therapeutic effect [60]. Therefore, in the present long-term stability study, the hardness (quantified through penetration degree) and spreadability of the OEO-PbH formulation were monitored and measured at pre-established times during the 6 month period under storage conditions. There were no significant differences between the hardness and spreadability data produced by the OEO-PbH formulation during the stability testing period. However, it must be noted that the results of the two rheological tests can be attributed to a very slight decrease in the formulation consistency over time at room temperature.

In a previous paper, our research group demonstrated that OEO-PbH elicited anti-proliferative, cytotoxic, anti-migratory, and pro-apoptotic effects against HaCaT human keratinocytes [51]. Furthermore, as revealed by the in ovo assessment using the CAM assay, our research team concluded that OEO-PbH was well tolerated and did not cause irritation symptoms compared to sodium lauryl sulfate used as an irritating solution. Moreover, the angiogenic process was modulated at the tested concentration by limiting the interconnection and dilation of the capillaries [50]. As FP composition includes keratinocytes and small blood vessels, the obtained effects of OEO-PbH lead to the idea of removal of skin tags in a non-invasive manner, thus to increase the patient’s compliance and life quality. Therefore, the results obtained in vitro and in ovo led to a further evaluation of the in vivo effects of this innovative formulation. 

Because of the strong irritant effect of EOs, the micellar encapsulation method was used in order to assure the safety of the product and to decrease the skin irritation caused by pure EOs [61,62]. For topical formulations based on polymeric micelles loaded with EOs, these concentrated multicomponent, hydrophobic liquid mixtures are encapsulated in the inner core of the micelles, thus being protected from degradation under different environmental conditions (e.g., temperature, light, pH, and oxygen) and promoting their penetration in and through the skin stratum corneum. Accordingly, the EOs’ bioavailability at the site of action increases and thus enhances their efficacy, which allows one to reduce the dose per surface unit and consequently to diminish local (skin) irritation [63]. 

The use of non-invasive methods for the evaluation and quantification of a series of skin parameters offered new perspectives in the clinical research area and enriched the scientific field due to the easiness, safety, and precision of these measurements. Some of the most common non-invasive methods currently used are corneometry, pH-metry, mexametry, tewametry, cutometry, and sebumetry [64,65]. As mentioned before, within this study, mexametric, corneometric, and tewametric measurements were conducted in order to evaluate and characterize the morphological and structural changes in skin tags after treatment with the tested formulation.

Mexametric measurements contribute to the evaluation of cosmetic, cosmeceutical, and dermatological treatments by measuring the concentration of melanin and hemoglobin in the skin [66]. Melanin is significantly increased in hyperpigmentation cases, such as sun, post-acne, and aging spots, as well as in malignant skin tumor development. On the other hand, increased hemoglobin levels are representative of irritant and sensitized skin. Thus, this parameter depicts information about the erythema index and is relevant in order to characterize inflammatory skin disorders (psoriasis, acne, and atopic dermatitis) [67,68]. The mexameter is the non-invasive device used to measure the skin concentration of the two abovementioned pigments, by means of light absorption. The light emitted by the probe is reflected by the skin and captured and measured by the receiver. The specific wavelengths for erythema measurement are 568 nm and 660 nm, corresponding to the spectral absorption peak of hemoglobin, while melanin levels can be measured at 660 nm and 880 nm [69]. 

Skin hydration is commonly characterized by two parameters, namely the water holding capacity of the stratum corneum, also known as skin surface water content (SWC), and the loss of transepidermal water (TEWL), with both features being beneficial in order to appreciate the effects of dermato-cosmetic treatments or to appraise several skin conditions. A dysfunctional skin process is highlighted by a decline in SWC and an increase in TEWL [70,71]. The corneometer measures the fluctuations in water content in the stratum corneum, expressed as SWC, via the determination of the dielectric constant of the skin. The measurement is performed at a depth of 10–20 μm in the stratum corneum, without the deeper skin layers influencing the skin hydration values [72]. The tewameter probe is equipped with two pairs of sensors which rate the density gradient of water evaporation with the help of the relative humidity and skin temperature. Therefore, TEWL represents the amount of condensed water that diffuses along the stratum corneum to the surface of the skin and is the most frequently used parameter for the evaluation of skin functionality (e.g., skin barrier function) in healthy skin as well as in skin dysfunctions [73,74]. 

The effects of OEO-PbH on skin tags were monitored both macroscopically and microscopically, while the changes in some physiological skin parameters were appreciated using non-invasive measurements. Within this evaluation, some of the skin parameters (erythema, SWC, and TEWL) that could give us relevant information regarding the effect of OEO-PbH on FPs were assessed. The non-invasive measurements confirmed an increase in the irritation degree and in TEWL, while SWC decreased during the eight weeks of evaluation compared with the normal values of these skin parameters measured in the first meeting with the subjects and also with those provided in the literature [75,76].

As a result of the erythema evaluation, OEO-PbH can be considered to be safe for human administration. Products with a strong irritative effect will be conducted to a significantly increased erythema index (the instrument can measure erythema values up to 1000 units) [77]. According to Firooz et al., the average normal values for erythema index, TEWL, and hydration are around 303.63 ± 100.73, 9.52 ± 7.36, and 49.06 ± 16.09 A.Us. in the case of females, and 378.14 ± 124.50, 15.49 ± 11.47, and 48.42 ± 22.12 A.Us. for males [76], respectively. Although we observed a growing trend for the first two studied parameters and a decreasing one for hydration level, there was no worrying difference between the values obtained before applying OEO-PbH and those determined in the last session of non-invasive measurements. 

The macroscopic evaluation of the FPs and the surrounding area after the application of OEO-PbH revealed that the FPs dried and shrunk, without visibly affecting the area around them. The feedback of the subjects was also essential for the evaluation of the hydrogel effect. From a clinical standpoint, a decrease in the percentage of lesions in the treated area could be observed, namely, after the first month of treatment it was approximately 20–30%, and after the second month of treatment it was approximately 50%, respectively. Lesions bigger than 5 mm were not influenced by the applied substance throughout the two months of treatment. The skin of the treated area did not present any changes after one month of treatment, but presented mild xerosis in 70% of the cases after two months, which rapidly subsided in 100% of cases after the application of emollient products. Erythema or descuamation were not clinically evident throughout the treatment. Other signs of adverse effects were not found upon clinical examination. Symptomatically, after the first month of treatment, 20% of patients reported mild recurrent pruritus (itching), especially after showering, which affirmatively remitted shortly after applying emollient topical products. This symptom usually accompanies xerosis [78]. None of the subjects experienced redness, flaking of the treated area, or other signs or symptoms of side effects throughout the treatment. 

Xerosis, also known as dry skin, xeroderma, or xerosis cutis, is determined by structural and functional changes in keratinocytes due to an imbalance between hydrophilic and lypophilic content [79]. Generally, there is a decrease in water and lipid components in the stratum corneum, and consequently a decrease in stratum corneum hydration and an increase in TEWL [80]. The symptoms of this very common dermatological condition include pruritus and a feeling of tightness. Patients may also present aching and burning symptoms in cases of advanced xerosis. Clinically, the skin is dry, harsh, scaly, and inflamed, and presents poor elasticity and a changed texture. Moreover, wrinkles, erythema, splits, and excoriations may also occur in moderate to severe xerosis, and thus an increased erythema index may also be obtained [81,82]. OEO-PbH produced mild xerosis on the treated area, a desired and useful symptom in the context of skin tag removal. A histological examination of the unfallen FPs was further conducted.

Histopathological examination of FPs adds a significant layer of understanding to the underlying mechanisms through which OEO-PbH exerts its beneficial effects on skin tags. The initial observations from untreated FPs provided a baseline for comparison, revealing a standard composition with a central core of fibro-collagenous tissue enveloped by a thin layer of the epidermis. Following one month of topical treatment with OEO-PbH, microscopic examinations reported an intriguing finding: a thinner epidermis in most cases, coupled with a more edematous dermis and a substantial influx of inflammatory cells. These histological alterations suggest that OEO-PbH may elicit anti-inflammatory responses and also affect the thickness of the epidermis. This is in line with our in vitro studies which demonstrated the anti-proliferative and pro-apoptotic effects of OEO-PbH on keratinocytes [51]. A reduction in epidermal thickness may potentially contribute to the macroscopic observation of skin tag shrinkage. The effects become more pronounced after two months of topical treatment with OEO-PbH. Hence, the fibrovascular cores of the FPs exhibited the most substantial changes. The core thickness was reduced, the size and number of blood vessels decreased, and cellularity was low. These alterations suggest that OEO-PbH can influence the fibrous core of FPs, leading to a reduction in size and changes in the blood supply to the FPs. This also confirms our previous in ovo results [50], where OEO-PbH modulated angiogenesis by limiting capillary interconnection and dilation. As FPs contain small blood vessels, affecting their structure and function may contribute to the non-invasive removal of skin tags. In conclusion, the microscopic examination of FPs at various time points suggests that topical treatment with OEO-PbH may indeed induce histological changes in FPs. These changes encompass alterations in the epidermis, dermis, and fibrovascular cores, contributing to an observed reduction in the size of the FPs after treatment with OEO-PbH. Moreover, this evidence supports the efficacy and potential of OEO-PbH as a therapeutic agent for skin tag management, offering a novel, non-invasive approach for enhancing patient compliance and life quality. Further investigations are needed to corroborate these findings and fully elucidate the molecular mechanisms behind the observed effects.

## 4. Materials and Methods

### 4.1. Materials

To obtain the poloxamer-based binary hydrogel loaded with OEO, oregano essential oil previously extracted from the species *O. vulgare* var. *vulgare* and characterized [51], Poloxamer 407 (trade name Pluronic^®^ F127), Pluronic L 31 purchased from Sigma-Aldrich-Chemie (Steinheim, Germany), and purified water were used. 

### 4.2. Preparation of Poloxamer-Based Binary Hydrogel Loaded with OEO

The polymeric-micelle-based hydrogel loaded with 5% *w*/*w* OEO was prepared via the “cold method” [83], as previously discussed in our in vitro evaluation [51] and in ovo [50] studies. Briefly, 20% *w*/*w* Pluronic F127 (P127) and 1% *w*/*w* Pluronic L 31 (P31) were slowly dispersed in cool (4 °C) purified water. The obtained dispersion was kept at 4 °C (refrigerator) for at least 24 h, stirring from time to time until the complete dissolution of poloxamers, indicated by the transparency of the formed solution. OEO in concentration of 5% *w*/*w* was added to the cold solution of poloxamers, while continuously stirring at room temperature, and a clear hydrogel was formed.

### 4.3. Quality Control Tests for OEO-PbH

#### 4.3.1. Long-Term Stability Evaluation of OEO-PbH

After being physicochemically characterized at 24 h after preparation through visual examination, pH, and consistency determination [51], the polymeric-micelle-based hydrogel loaded with 5% *w*/*w* OEO was packed in an amber tightly closed glass jar, and stored at room temperature (25 ± 2 °C) for six months. Throughout the stability study over time, the OEO-PbH formulation was assayed for organoleptic characteristics, pH, OEO content, and consistency on days 7, 14, 28, 60, 90, and 180.

Visual examination was performed to observe the possible changes over time in organoleptic characteristics (transparency, color, odor, homogeneity, and consistency).

For pH determination, the hydrogel was previously diluted in water to a concentration of 5% (*m*/*v*) and stirred for 15 min at room temperature. Then, the potentiometric analysis [84] of the obtained clear solution was performed using a pH-meter (Sension™ 1 portable digital pH meter, Hach Company, Columbus, OH, USA) in triplicate, at 25 ± 2 °C.

The OEO content in the sample was determined throughout the stability study via ultraviolet–visible (UV-VIS) spectrophotometry at 216 nm (T70 + Spectrophotometer, PG Instruments Limited, Lutterworth, U.K.), according to the methodology described below. A total of 0.200 g hydrogel sample (equivalent to 10 mg OEO) was accurately weighted and transferred to a 10 mL glass amber volumetric flask; then, it was diluted with ethanol 96% (*v*/*v*) to 10 mL. After adequate dilution, the absorbance of the ethanolic solution was measured in 1 cm quartz cells at room temperature. The determination of OEO content was repeated three times for each experiment. The OEO content of the samples was calculated using the following equation, describing the calibration curve of OEO: y = 0.72x + 0.0372 (= 0.9996), where y is the absorbance at 216 nm and x is the OEO concentration (mg %); the value of the correlation factor, R^2^, was 0.9996. 

The consistency of the OEO-PbH formulation was evaluated through two related parameters, namely penetration degree and spreadability, which were measured via the penetrometric method and parallel-plate method, respectively. The penetrometry measurements were carried out using a penetrometer equipped with a penetrating microcone (PNR 12, Petrolab, Speyer, Germany) following the procedure described by the European Pharmacopoeia [85]. The spreadability tests were conducted using the del Pozo Ojeda-Suñé Arbussá extensometer and according to the method described in the literature [86]. The consistency measurements were performed in triplicate at 25 ± 2 °C.

#### 4.3.2. Sterility Testing

For microbial contamination control, the total count of the CFUs of aerobic microorganisms (bacteria, fungi) and the absence of pathogenic or conditionally pathogenic microorganisms (*Salmonella* spp., *Escherichia coli*, *Pseudomonas aeruginosa*, *Staphylococcus aureus*) were determined. The testing method and the interpretation of the results were performed according to the European Pharmacopoeia, 10th Edition [87].

The working material was represented by 10 g of the tested formulation, diluted in ethanol (EtOH) 10%. The lack of anti-microbial activity for EtOH 10% was tested using the disk-diffusion method. Blank paper disks (Biomaxima, Poland), that 15 µL of EtOH was pipetted onto, were placed on the surface of a Mueller-Hinton agar, pre-inoculated with a 0.5 Mc Farland microbial suspension of reference strains (*Escherichia coli* ATCC 25922, *Staphylococcus aureus* ATCC 25923, *Pseudomonas aeruginosa* ATCC 27853, *Candida parapsilosis* ATCC 22019). The plates were incubated at 35 °C for 24 h.

To determine the total count of CFU, 1 mL of the 1/10, 1/100, and 1/1000 dilutions was pipetted onto sterile Petri dishes; then, 15 mL of melted agar (simple agar for bacteria or Sabouraud for fungi) was added, at a temperature of approximately 45 °C. After homogenizing the contents, the plates were left at room temperature until the agar solidified, and then they were incubated accordingly (48 h at 35 °C for bacteria and 7 days at 25 °C for fungi). To determine the total count of CFU of coliforms, 1 mL of each dilution (1/10, 1/100, and 1/1000) was added to 10 mL of Mac Conkey broth and lactose broth, respectively (2 series with 3 tubes/dilution); then, the tubes were incubated at 37 °C and 45 °C for 48 h. To identify *Salmonella* spp., 10 mL of the 1/10 dilution of the tested sample was suspended in 100 mL of selenite broth, incubated at 37 °C for 24 h, and then re-inoculated on the Istrati-Meitert solid media. After incubation, the medium-colored (blue-green) or black colonies, characteristic of *Salmonella* spp., were absent. For the identification of *Escherichia coli*, 10 mL of the 1/10 dilution of the tested sample was suspended in 100 mL of lactose broth, incubated at 45 °C for 24 h, and then re-inoculated on Mac Conkey solid media. After incubation, the lactose-positive (red) colonies characteristic of *Escherichia coli* were absent. For the identification of *Pseudomonas aeruginosa*, 10 mL of the 1/10 dilution of the tested sample was added to 100 mL of 2% glucose broth, incubated at 37 °C for 24 h, and then re-inoculated on the Istrati-Meitert solid media and on *Pseudomonas* agar. After incubation at 37 °C for 24 h, the characteristic colonies of *Pseudomonas* spp. (smell, pigment) were absent. In 100 mL of Chapman broth, 10 mL of the 1/10 dilution of the tested sample was inoculated and incubated at 37 °C for 24 h, and then re-inoculated on Columbia agar +5% sheep blood (COS) and on Chapman agar. After incubation at 37 °C for 24 h, cream-yellow colonies on COS, often with hemolysis, or yellow colonies associated with a change in the color of the media in yellow, characteristic of *Staphylococcus aureus*, were absent.

#### 4.3.3. Preservative Efficacy Test (PET)

For the PET, the microbial strains were selected according to the European Pharmacopoeia: *Staphylococcus aureus* ATCC 6538P, *Escherichia coli* ATCC 8739, *Pseudomonas aeruginosa* ATCC 9027, *Candida albicans* ATCC 10231, and *Aspergillus niger* ATCC 16404 (Merck Group, St. Louis, MO, USA). 

The microbial suspension was prepared at approximately 10^8^ microorganisms/mL for all of the tested strains. A total of 0.1 mL from each suspension was inoculated in 20 g of the tested product, resulting in a final microbial concentration of 10^5^–10^6^ microorganisms/g. After incubation at 25 °C, 1 g of the inoculated product was dispersed on the surface of the culture media (Columbia agar +5% sheep blood for all bacterial strains, Chapman agar for *Staphylococcus aureus*, Mac Conkey agar for Gram-negative bacteria, and Sabouraud for fungi) immediately and at 6 h, 24 h, and 48 h after inoculation of the tested product. 

### 4.4. Study Design

A total of twenty volunteers who met the eligibility criteria were recruited by the dermatologist and enrolled in the study. This study was approved by the research ethics committee of the Victor Babes University of Medicine and Pharmacy Timisoara, Romania (Nr. 04 a/17 June 2022), and complied with the rules of good practice in biomedical research and with the principles established by the Declaration of Helsinki [88]. Some inclusion and exclusion criteria were also established. Subjects with dermatological disorders or cutaneous lesions near the FP, who applied topical treatment to the area of interest, with an allergy to hydrogel ingredients, pregnant women, oncological patients, and people over seventy years of age, as well as minors, were excluded from the study. The subjects were informed and instructed about the study, and they approved and signed the informed consent. The initial evaluation was carried out to appreciate the medical history of the subjects, to perform the objective examination, to evaluate the skin through dermoscopy and Wood lamp to establish a positive diagnosis of FP, and to conduct a series of non-invasive determinations on the FP area before starting the application of the hydrogel. 

At the initial dermatology consultation, one FP was removed from each participant and processed in the pathology laboratory (this was time frame 0). Afterward, the subjects applied the test product (OEO-PbH) twice a day for two months on the identified FPs. Patients were instructed to apply a small pea-sized amount of the tested product using a plastic applicator (received together with the OEO-PbH formulation) on the identified FPs. The study was conducted over eight weeks, and during which, the subjects came to the dermatologist’s office every two weeks for a non-invasive evaluation of the FP area. The following skin parameters were evaluated: erythema index, TEWL, and SWC. After one month of treatment with OEO-PbH, a second FP was removed from each participant and processed in the pathology laboratory (this was the time frame of 30 days). After two months of treatment with OEO-PbH, a third FP was removed from each participant and processed in the pathology laboratory (this was the time frame of 60 days). The purpose of these evaluations was to observe whether there were changes in FP composition and structure at the histological level.

### 4.5. Skin Parameter Measurements

Three physiological parameters were evaluated in order to assess the skin response to OEO-PbH by means of non-invasive methods previously described by Simu et al. [89]. A Multiprobe Adapter System (MPA5) from Courage-Khazaka Electronics, Germany, equipped with a Mexameter^®^MX18 probe, Tewameter^®^TM300 probe, and Corneometer^®^CM 825 probe, respectively, was used to evaluate erythema index, TEWL, and SWC, respectively. All of the measurements were performed using the same operator, in predefined temperature (20–23 °C) and humidity conditions (40–60%). The subjects were asked to wait for at least 15 min in the test room before starting the experimental part for the skin to adapt to the indoor environment. Measurements were taken in the same tested area and performed in triplicate, further using the average values.

### 4.6. Histological Processing 

After the FPs were removed from each of the twenty participants in the study, they were sent to the pathology lab for histological analysis. After grossing and histoprocessing, the paraffin-embedded FPs were then cut into thin sections, typically between 4 and 6 microns thick, using a microtome. The sections were placed on glass slides and then stained with H&E stain, which highlights the nuclei and cytoplasm of cells in the tissue. The H&E-stained slides were then examined under a microscope in order to evaluate the FPs for various histological features. This included assessing the composition of the fibrous core, the presence of hyperplasia or papillomatosis in the overlying epidermis, and the presence of other histological changes such as keratotic cysts or pigment in basal epidermal keratinocytes. This process was repeated for all twenty participants in the study, with three FPs per participant being analyzed histologically at different time points (at the initial dermatology consultation and after 30 and 60 days of treatment with OEO-PbH).

### 4.7. Statistical Analysis

GraphPad Prism 8.01 software (San Diego, CA, USA) was used in order to evaluate the measurements. Data were represented as mean ± SD and the results were analyzed by means of one-way ANOVA and Dunnett’s multiple comparison test (* *p* < 0.05; ** *p* < 0.01; *** *p* < 0.001; **** *p* < 0.0001).

## 5. Conclusions

In our previously published studies, the successful formulation and in vitro [51] and in ovo [50] characterization of a poloxamer-based binary hydrogel loaded with 5% oregano essential oil were described.

In the present evaluation, the long-term stability profile, the susceptibility to microbial contamination, and the in vivo tolerance of this OEO-loaded polymeric micelle hydrogel were evaluated. The physicochemical stability of this formulation under the tested conditions (6 months at room temperature) was highlighted. No change in organoleptic properties, pH, OEO content, or consistency of the Pluronic F 127/L 31 binary hydrogel loaded with 5% OEO was observed. The consistency tests (penetrometry and spreadability) confirmed that the two key rheological attributes promoting patient acceptability and therapeutic efficacy (prolonged residence time on the treated area and ease of application to the skin) were maintained after 6 months of storage at room temperature. Moreover, the hydrogel showed a lack of microbial contamination. The two-month-long clinical study (supported by both the non-invasive measurements as well as the histopathological examination) showed that OEO-PbH can be a therapeutic option for FPs smaller than 5 mm. This study, together with the previous ones quoted within this manuscript, offers a comprehensive scientific picture of a non-invasive modern pharmaceutical formulation for the management of FPs.

## Data Availability

Data is contained within the article.

## Abbreviations

ANOVA	Analysis of variance
A.U.	Arbitrary units
CAM	Chorioallantoic membrane
CFU	Colony-forming units
COS	Columbia agar +5% sheep blood
EO	Essential oil
EtOH	Ethanol
FP	Fibroepithelial polyp
H&E	Hematoxylin and eosin
HaCaT	Human keratinocyte cell line
HDL	High-density lipoprotein
IGF-1	Insulin-like growth factor-1
IL-6	Interleukin-6
IL-10	Interleukin-10
IL-23	Interleukin-23
OEO	*Origanum vulgare* L. essential oil
OEO-PbH	*Origanum vulgare* L. essential oil-poloxamer-based hydrogel
PET	Preservative efficacy test
SD	Standard deviation
SWC	Skin surface water content
TEWL	Transepidermal water loss
TNF-α	Tumor necrosis factor
UV-VIS	Ultraviolet-visible
